# Applicability of Stress Cardiac Magnetic Resonance Imaging in Patients With Cardiac Implantable Devices: A Systematic Review

**DOI:** 10.1155/crp/3131535

**Published:** 2026-06-28

**Authors:** Sarina Zakavi, Mohammad Rafie Khorgami, Pedram Babaee, Alireza Babaeizadeh, Rahem Rahmati, Mina Felfeli, Elahe Meftah, Golnaz Houshmand, Fatemeh Abedpour, Amirfarjam Fazelifar, Hamidreza Pouraliakbar, Hamidreza Hekmat, Negar Omidi

**Affiliations:** ^1^ School of Medicine, Tehran University of Medical Sciences, Tehran, Iran, tums.ac.ir; ^2^ Tehran Heart Center, Cardiovascular Diseases Research Institute, Tehran University of Medical Sciences, Tehran, Iran, tums.ac.ir; ^3^ Rajaie Cardiovascular Medical and Research Center, Iran University of Medical Sciences, Tehran, Iran, iums.ac.ir; ^4^ Department of Cardiology, School of Medicine, Hajar Hospital, Shahrekord University of Medical Sciences, Shahrekord, Iran, skums.ac.ir; ^5^ Department of Anesthesiology, School of Medicine, Hajar Hospital, Shahrekord University of Medical Sciences, Shahrekord, Iran, skums.ac.ir; ^6^ Students Research Committee, Shahrekord University of Medical Sciences, Shahrekord, Iran, skums.ac.ir; ^7^ Department of Internal Medicine, Brookdale University Hospital Medical Center, Brooklyn, USA, brookdalehospital.org; ^8^ Students’ Scientific Research Center (SSRC), Tehran University of Medical Sciences, Tehran, Iran, tums.ac.ir; ^9^ School of Medicine, Baharloo Hospital, International Campus, Tehran University of Medical Sciences, Tehran, Iran, tums.ac.ir

**Keywords:** cardiovascular imaging, CIED, CMR, stress CMR, systematic review

## Abstract

**Purpose:**

The utilization of stress perfusion cardiac magnetic resonance (CMR) in patients with cardiac implantable electronic devices (CIEDs) is still limited.

**Methods:**

The study was registered in the Prospective Register of Systematic Reviews (PROSPERO) with ID CRD42023457308. PubMed, Scopus, Embase, Web of Science, ProQuest, and CINAHL databases were searched using the Mesh and related terms of CMR imaging, stress, and CIEDs.

**Results:**

Out of the 1695 papers we found, eight met our inclusion criteria. We reviewed the included studies and provided a concluding remark concerning (a) image quality; (b) CMR compatibility, scanner, and safety; (c) device protocols and exclusion criteria; (d) vasodilator choices, effects, and safety; and (e) clinical outcomes.

**Conclusion:**

This study has demonstrated a positive trend in the utilization of stress CMR in MR‐conditional devices. However, the review has identified multiple research gaps that warrant further investigation.

## 1. Introduction

Stress cardiovascular magnetic resonance (CMR) is a noninvasive multiplanar imaging modality that can evaluate myocardial ischemia, viability, and function. Due to its outstanding diagnostic accuracy, stress CMR has been widely implemented for evaluating confirmed or suspected patients with coronary artery disease (CAD) [1].

Among noninvasive imaging modalities for myocardial ischemia, stress CMR is recognized for its high diagnostic performance, validated against the reference standard of invasive fractional flow reserve (FFR) and supported by clinical guidelines [2, 3]. Its high spatial resolution and wide field of view uniquely allow CMR sequences to eliminate attenuation artifacts, providing a robust assessment of myocardial perfusion and viability [4].

Stress CMR is based upon dynamic contrast‐enhanced perfusion imaging, which uses electrocardiogram‐gated fast T1‐sensitive imaging during stress or rest [5]. Pharmacological stress CMR is the typical approach to induce hyperemia by using both vasodilators and inotropic agents [6]. After achieving hyperemia, a gadolinium‐based contrast agent (GBCA) is administered, and serial T1‐weighted CMR images are captured [4]. Then, either stopping the vasodilator or administration of a pharmacologic agent may be selected for reversal of hyperemia. Shortly after, a series of cine CMR images is obtained. Resting perfusion images subsequently commence by employing the identical technique and slice. For assessing myocardial viability, late gadolinium enhancement (LGE) images are captured after a 5‐min latency to remove the GBCA from healthy myocardium [7]. The presence of LGE was adjudicated on the basis of visible myocardial enhancement observed in either cross‐cut or phase swap magnetic resonance images [8].

Potential contraindications for a CMR scan must be thoroughly evaluated in patients referred for it. Due to artifacts of devices with ferromagnetic materials that impair image interpretability, patients with these devices may not be the appropriate applicants for stress CMR [4]. Therefore, wearing metallic devices is a contraindication to CMR studies. These metallic devices include ocular metallic bodies, vascular (particularly cerebral) clips, and implanted pumps for medication infusion [9]. However, most modern cardiac implantable electronic devices (CIEDs), including pacemakers and implantable cardioverter defibrillators (ICDs), are labeled as MR‐conditional and can be safely scanned cautiously if the manufacturer’s instructions are applied [7, 10]. Despite this, stress perfusion CMR utilization in patients with CIEDs is still limited, mainly to reflect the interacting potential of adenosine with cardiac pacing that may result in potential artifacts [11, 12].

Several studies support the safety [13, 14] and sufficient diagnostic image quality of CMR in patients with CIEDs [15–17]. Otherwise, the available literature remains limited to 1.5‐T (T) scanners and noncardiac 3‐T investigations [18, 19]. Besides, few studies have examined adenosine stress‐CMR in individuals implanted with MR‐conditional devices. The aim of the present study was to systematically evaluate the image quality, diagnostic utility, device protocols, vasodilator efficacy, and safety of the available stress CMR studies in patients with CIED. We also assessed the studies concerning potential magnetic field interferences with permanent pacemakers (PPM) and ICD devices after the CMR scan.

## 2. Methods

### 2.1. Search Strategy

We adhered to Preferred Reporting Items for Systematic Reviews and Meta‐Analyses (PRISMA) guidelines in this systematic review (Supporting Table 1). The study was registered with the Prospective Register of Systematic Reviews (PROSPERO) under the ID CRD42023457308. Three independent reviewers conducted a comprehensive search of PubMed, Embase, Scopus, Web of Science, ProQuest, and CINAHL for all relevant articles without any restrictions on the publication date and language. Group discussion was considered for resolving the discrepancies between the reviewers.

We searched the literature using the MeSH and related terms of implantable defibrillators and pacemakers, stress and vasodilators, and CMR imaging to find related papers. The specific search syntax for each term is provided in Supporting Table 2. The search was last updated on June 29, 2024. Additionally, manual screening of the reference lists of relevant studies was performed to detect any potentially relevant articles.

### 2.2. Study Selection and Data Extraction

After removing duplicates, three independent reviewers screened the titles, abstracts, and full texts of the identified articles. We included studies meeting all the following criteria: (a) peer‐reviewed original studies; (b) involved patients with a CIED implanted at least 6 weeks before imaging; and (c) stress CMR imaging was performed.

Meeting any of the following criteria led to the exclusion of the study: (a) chest x‐ray (CXR) showed evidence of abandoned, epicardial, or fractured leads; (b) cerebral clips or metallic eye implants; (c) age < 18; (d) contraindications to adenosine or dipyridamole (such as severe asthma or chronic obstructive pulmonary disease); (e) an allergy to GBCA; and (f) glomerular filtration rate (GFR) of less than 30 mL/min/1.73 m^2^.

### 2.3. Quality Assessment

Two reviewers independently evaluated the methodological quality of the included studies using the widely recognized tool for assessing observational research, the Newcastle–Ottawa Scale (NOS) [20, 21], and the Joanna Briggs Institute Critical Appraisal tools for use in systematic reviews—the checklist for case reports [22]. Any discrepancies between the reviewers were resolved through discussion, with a third author serving as an arbiter to reach consensus.

## 3. Results and Discussion

### 3.1. Search Results and Included Studies

**FIGURE 1:**
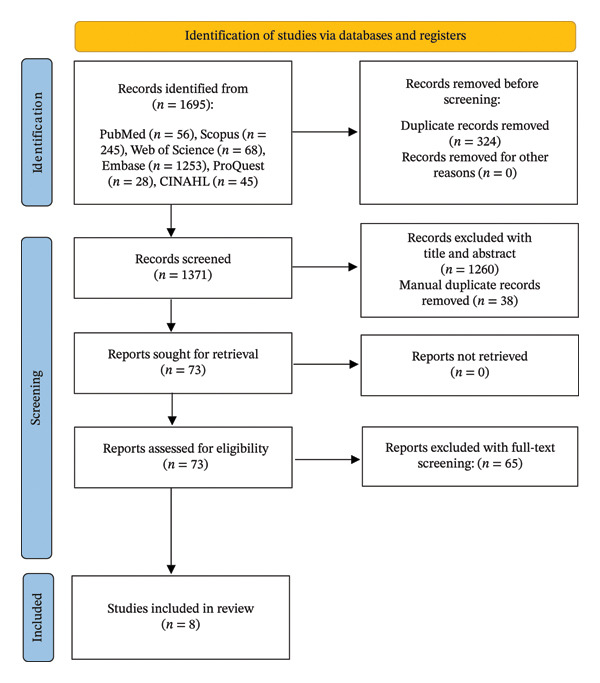
The PRISMA flow diagram.

In total, we found 1695 papers in the manual search of the articles’ citations, PubMed, Embase, Scopus, Web of Science, ProQuest, and CINAHL databases. Among them, eight papers were included in our study. The PRISMA flow diagram is depicted in Figure 1. Table 1 highlights the characteristics of the included studies. In our quality assessment, all studies received a score ≥ 7 (Supporting Table 3). The studies were assessed in five categories.

**TABLE 1:** Characteristics of included studies.

Study	Year	Number of patients (stress CMR)	Aim of study	Study design	Type of vasodilator (*n*)	Type of CIED and MRI	CIED settings during CMR	Outcomes
Klein‐Wiele et al. [12]	2015	24 (24)	Feasibility and safety of stress CMR in patients with MR‐conditional PMs	Retrospective Single center	Adenosine (24)	100% MR‐conditional PMs All in 1.5 T CMR scanner (ESPREE—Siemens Healthcare, Erlangen, Germany)	All devices were set to manufacturer MR‐safe mode. Five patients set to asynchronous pacing, 17 to ODO, 1 to AOO. No patient was PM‐dependent.	HR increased significantly by 12.3 ± 8.3 bpm (*p* = 0.001) but remained constant in AV‐block patients. No bradycardia occurred. Asynchronous pacing above resting HR did not interfere with intrinsic rhythm. Considering HR response to adenosine is important in choosing pacing mode during stress CMR.
Klein‐Wiele et al. [23]	2016	61 (59)	MR‐conditional PM‐related artifacts on quality of stress CMR	Retrospective	Adenosine (59)	100% MR‐conditional PMs All in 1.5 T CMR scanner (ESPREE–Siemens Healthcare, Erlangen, Germany)	All devices were set to manufacturer MR‐safe mode (pacing mode (DOO/AOO/VOO/ODO) was chosen based on the current intrinsic rhythm). Seven patients were PM‐dependent.	No PM‐induced artifacts in perfusion, flow, or T1w. Right‐sided devices caused no artifacts. In left‐sided devices, SSFP cine had more artifacts than LGE (*p* < 0.001). Valve and STIR sequences rarely affected. No adverse events. Device parameters were unchanged post‐CMR except a small, statistically significant decrease in ventricular pacing threshold (*p* = 0.027) deemed not clinically relevant.
Klein‐Wiele et al. [11]	2017	47 (47)	Pacing mode (deactivation vs. asynchronous pacing) during stress CMR of patients with MR‐conditional PM	Prospective single center	Adenosine (47)	100% MR‐conditional PMs All in 1.5 T CMR scanner (ESPREE—Siemens Healthcare, Erlangen, Germany)	All devices were set to manufacturer MR‐safe mode. Sixteen patients were set to asynchronous pacing. Eight patients were PM‐dependent.	During stress CMR, mandatory PM stimulation was differentiated from deactivation; moreover, asynchronous pacing did not interfere with intrinsic heart rhythm, and there was no competitive stimulation. Preserved AV conduction was found in 10 of 15 (66.7%) patients with intermittent AV block despite receiving adenosine. Pacing deactivation was safe in stress CMR of SND and AF patients. No bradycardia was observed.
Lindemann et al. [24]	2020	208 (27)	Clinical utility of CMR in patients with ICD presenting with electrical instability or worsening HF symptoms	Retrospective single center	Adenosine (27)	45% MR‐conditional ICDs 1.5 T CMR scanner (Ingenia, Philips Healthcare, Best, the Netherlands)	MR‐conditionals set to manufacturer MR‐safe mode. Non‐MR conditionals were set to pacing off, sensing‐only (ODO or OVO), or asynchronous pacing (VOO) based on the patient’s intrinsic rhythm.	CMR changed diagnosis in 40% and treatment in 21% of patients. High diagnostic image quality achieved.
Miller et al. [25]	2022	67 (19)	Hemodynamic response and safety of vasodilator CMR vs. SPECT in patients with permanent PMs or ICD	Retrospective single center	Adenosine (12) Regadenoson (7)	90% MR‐conditional PPMs or ICDs All in 1.5 T CMR scanner (MAGNETOM Aera), except 3 in 3.0 T system (Vida, Siemens Healthineers)	50% of patients were set to asynchronous pacing. The rest are set to deactivate (to OOO, OVO, OAO, or ODO programs).	Patients with CIED programmed to asynchronous pacing experienced no change in HR with vasodilator stress, and one study was aborted following the perfusion localizers (before vasodilator agent) due to excessive artifact in CMR of nonconditional ICD. Patients with deactivated CIED had HR elevations of a median of +18 (14–21) beats per minute (*p* = 0.01) and a significant decrease in SBP. DBP decreased significantly in all 19 patients. Stress CMR segments were diagnostic in the majority of cases with nonconditional ICDs causing 40 of 57 (70%) of nondiagnostic segments.
Motazedian et al. [26]	2022	1	Evaluating myocardial viability via stress CMR in a patient with an MR‐conditional PM with a 3.0 T system	Case report	Adenosine (1)	MR‐conditional PM in 3.0 T CMR scanner (Siemens Prisma 3‐T scanner, Munich, Germany)	The device was set to manufacturer MR‐safe mode (SureScan), and the patient was not PM‐dependent.	Stress CMR with a 3‐T scanner was safe and successful in diagnosing myocardial ischemia in a patient with CIED. Image quality was high and was not compromised despite the presence of CIED.
Pavon et al. [17]	2022	66 (66)	Feasibility of stress CMR in patients with MR‐conditional transvenous PPMs and defibrillators	Retrospective single center	Adenosine (66)	100% MR‐conditional PPMs or ICDs All in 1.5 T CMR scanner (MAGNETOM Aera or Sola, Siemens Healthineers, Erlangen, Germany)	All devices were set to manufacturer MR‐safe mode. Fifty patients were set to continuous pacing in asynchronous mode due to > 1% atrial or ventricular pacing (17 in VOO and 33 in DOO). Sixteen did not need pacing (ODO).	HR and SBP did not change during stress CMR, while DBP decreased significantly (*p* = 0.007). Image quality was diagnostic in 98% of cases, with positive ischemia in 9% of patients, all confirmed with CAG. Immediately and 1 year after CMR, there was no reported device damage or malfunction.
Pezel et al. [27]	2023	304 (304)	Safety, feasibility, and prognostic value of stress CMR in patients with MR‐conditional PMs	Retrospective bicenter	Adenosine (107) Dipyridamole (267)^∗^	100% MR‐conditional PMs All in 1.5 T CMR scanner (MAGNETOM Avanto, Espree, and Aera, Siemens Healthcare, Erlangen, Germany)	Devices are set to manufacturer MR‐safe mode (DOO/VOO for PM‐dependent patients (defined by HR < 30/min) and VVI/DDI for non‐PM‐dependent patients). Eight patients were PM‐dependent.	Nine patients had inconclusive stress CMR, and 22 were lost to follow‐up, hence, omitted. 11.7% experienced MACE during the median of 7.1‐year follow‐up (5.4–7.5). Ischemia and LGE were significantly associated with and independent predictors for MACE. Image quality was good or excellent in 84.9% of segments, and stress CMR was tolerated with no significant change in lead threshold or pacing parameters.

*Note:* AV: atrioventricular; CV: cardiovascular; PM: pacemaker; PPM: permanent pacemakers; CAG: coronary artery angiography.

Abbreviations: AF, atrial fibrillation; CAD, coronary artery disease; CIED, cardiac implantable electronic devices;, CMR, cardiac magnetic resonance; DBP, diastolic blood pressure; HF, heart failure; HR, heart rate; ICD, implantable cardioverter defibrillator; LGE, late gadolinium enhancement; MACE, major adverse cardiovascular event; MI, myocardial infarction; MRI, magnetic resonance imaging; SBP, systolic blood pressure; SND, sinus node dysfunction; SPECT, single‐photon emission computed tomography.

^∗^70 patients excluded.

### 3.2. Heterogeneity and Methodological Variability

Significant methodological heterogeneity exists among the included studies, reflecting the evolving clinical practice of performing stress CMR in CIED patients [28]. This variability includes (1) scanner field strength (predominantly 1.5 T with limited 3 T data); (2) device type and conditionality (MR‐conditional pacemakers and ICDs, with scant evidence on non‐MR‐conditional devices); and (3) device programming strategies during pharmacological stress. These factors influence safety profiles, image quality, and hemodynamic responses and contribute to the observed variation in reported outcomes. As such, the subsequent synthesis of evidence is presented with these methodological differences in mind, and findings are contextualized accordingly to provide a nuanced interpretation of the aggregated data.

### 3.3. Image Quality

Image quality was evaluated based on the 17‐segment system of the American Heart Association regarding the artifacts and applicability for diagnosis. Six studies discussed image quality [17, 23–27]. A comparison of image quality findings across studies is presented in Table 2.

**TABLE 2:** Image quality.

First author (year)	Device type	Field strength	Key sequences assessed	% diagnostic segments/scans	Common artifacts and location	Notes on quality stratification	Ref
Klein‐Wiele (2016)	PPM (conditional)	1.5 T	SSPP, GRE (perfusion, LGE, STIR, flow)	Perfusion/Flow/T1w: 100% artifact‐free; LGE: 89.2%	SSPP cine (25% artifact rate). STIR valve sequences are affected by left‐sided devices.	Right‐sided devices: No artifacts. Sequence: GRE/LGE > SSPP for artifact reduction.	[23]
Lindemann (2020)	ICD (45% conditional)	1.5 T	Cine, LGE, T1/T2, perfusion, angio	98 ± 2.2%	Anterior LV segments (most common).	Included nonconditional ICDs; overall high diagnostic yield for perfusion.	[24]
Motazedian (2022)	PPM (conditional)	3 T	Cine (SSFP), perfusion, LGE	High‐quality, diagnostic	Minor inferolateral susceptibility artifact (SSFP only);RV lead artifact. Neither compromised diagnosis.	Single case report; demonstrates feasibility at 3 T with conditional device.	[26]
Pavon (2022)	PPM and ICD (conditional) • PPM: *n* = 36 • Transvenous ICD: *n* = 28 • SubQ ICD: *n* = 2	1.5 T	Cine (b‐SSFP or GRE), stress perfusion (GRE), LGE (PSIR‐GRE)	PPM: 97%. Transvenous ICD: 100% SubQ‐ICD: Cine images nondiagnostic in 100%, stress test not performed.	SubQ ICD: nondiagnostic cine. Transvenous ICD: artifacts in anterior wall.	Device‐specific: SubQ‐ICD problematic. Transvenous ICDs yielded diagnostic perfusion despite artifacts.	[17]
Miller (2022)	PPM/ICD (90% conditional)	1.5 T (*n* = 16), 3 T (*n* = 3)	Cine, perfusion, LGE	High diagnostic yield in conditional devices; nonconditional ICDs accounted for 70% of nondiagnostic segments	Anterior/anteroseptal walls (especially with nonconditional ICDs)	MR Label: Nonconditional devices major source of nondiagnostic segments. Limited 3 T data show potential challenges.	[25]
Pezel (2023)	PPM (conditional)	1.5 T	Cine (b‐SSFP or GRE), stress perfusion (b‐SSFP or GRE), LGE (3D IR‐GRE)	84.9% rated good/excellent	Artifacts more frequent with SSFP.	Side: right‐sided > left‐sided (*p* < 0.001). Sequence: GRE > SSPP (*p* < 0.001).	[27]

*Note:* PPM, permanent pacemaker; SSPP: steady‐state free precession; GRE: gradient echo; SubQ: subcutaneous.

Abbreviations: CIED, cardiac implantable electronic device; CMR, cardiac magnetic resonance; ICD, implantable cardiovarter‐defibrillator; LGE, late gadolinium enhancement; STIR, short tau inversion recovery.

In 2016, Klein‐Wiele et al. [23] investigated CIED artifacts in specific CMR sequences. They found that right‐sided implants caused no significant artifacts in any sequence. In left‐sided devices, first‐pass perfusion, flow analysis, and T1‐weighted images were free from artifacts, and LGE sequences had significantly fewer artifacts than steady‐state free precession (SSFP) cine sequences (10.8% vs. 25%, *p* < 0.001). While aortic valve, mitral valve, and STIR sequences showed compromised image quality in a minority of left‐sided cases (7.9%, 5.3%, and 8.6% of patients, respectively), the overall diagnostic utility of CMR was preserved.

Four years later, Lindemann et al. [24] reported that perfusion images were diagnostic for evaluating 98 ± 2.2% of left ventricular myocardial segments. Three years later in another study, Pezel et al. [27] found that the mean score for image quality was 4.3 ± 0.7 out of 5, with 84.9% of them scoring great/excellent. Nonetheless, gradient echo sequences (GRE) were obtained in 59.3% of patients due to significant device artifacts and yielded significantly better scores than balanced‐SSFP sequences (mean score of 4.5 ± 0.7 vs. 3.9 ± 0.7, respectively; *p* < 0.001). Also, right‐sided pectoral devices had better image quality than the left devices (mean score of 4.6 ± 0.5 vs. 4.1 ± 0.7, respectively; *p* < 0.001). Similar to the findings by Klein‐Wiele et al. [23] in 2016, they reported that right‐sided devices project no artifacts, irrespective of the sequences.

Three other studies regarding image quality were also published in 2022. Among them, Pavon et al. [17] used a different image scoring system, which was validated by Klinke et al. [29] in 2013, and designated images to Grades 4 (nondiagnostic), 3 (poor), 2 (moderate), and 1 (good). CMR was diagnostic if scored 1 to 3. They studied devices individually. In MR‐conditional PPMs, 97% of CMRs had good quality and were diagnostic, with one patient having to repeat the test due to not interpretable results because of the Valsalva maneuver and contrast injection into a thrombosed vein and two patients’ respiratory motions leading to moderate LGE quality. In MR‐conditional ICDs, a stress test was not done in two patients with subcutaneous ICDs because they had extensive artifacts with nondiagnostic cine images. All transvenous ICDs had diagnostic perfusion‐image quality with poor and moderate qualities due to device‐induced artifacts projecting into the anterior ventricular wall and only one poor‐quality image artifact caused by respiratory motion.

Furthermore, Miller et al.’s [25] findings were similar to previous authors’ in demonstrating the good diagnostic quality achievable with MR‐conditional devices. However, their study also provided preliminary insight into the challenges associated with non‐MR‐conditional devices. They performed CMR in two such devices (10% of their cohort) and had to abort one scan in a nonconditional ICD prior to adenosine administration due to excessive artifact. Their limited data suggested that nonconditional ICDs accounted for the majority (70%) of nondiagnostic segments, highlighting a significant concern that requires validation in larger, dedicated studies. In this study, three patients with 3‐T conditional devices were studied in 3‐T scanners, of whom one had an uninterpretable apical segment of the septum. However, Motazedian et al. [26] reported the first successful stress perfusion CMR case on one patient with a CIED in a 3‐T scanner and had high‐quality images without significant artifacts.

Overall, results from stress CMR scans for CIED patients show improved image quality over time. GRE typically produces higher‐quality images than balanced‐SSFP sequences, and there are fewer artifacts in specific sequences when using left‐sided devices. The image quality is better in right‐sided pectoral devices than in left‐sided ones. Challenges with artifacts in cine images persist for MR‐conditional ICD devices, and the percentage of nondiagnostic segments is higher in non‐MR‐conditional devices, given the artifacts. Patients using CIEDs, especially those with MR‐conditional devices, have undergone stress CMR with high‐quality images despite these challenges related to device‐induced artifacts and interpretability. Technological improvements have enhanced the diagnostic capabilities of CMR imaging. However, there are still certain limitations.

### 3.4. MR Compatibility, Scanners, and Safety

Evidence from various studies, including those by Pezel et al. [27], who studied 304 patients; Klein‐Wiele et al. [11, 12, 23], who studied 47, 59, and 24 patients, respectively; and Pavon et al. [17], with a population study of 66, indicates that patients with MR‐conditional devices can safely and effectively undergo vasodilator stress CMR. These studies, conducted on a total of 500 patients with MR‐conditional devices using 1.5‐T scanners, reported no death, major adverse cardiovascular event (MACE), or change in device integrity, lead impedance, pacing capture thresholds, or battery voltage pre‐ and post‐CMR.

In another study, Lindemann et al. [24] included 208 patients with ICDs, of which only 45% were MR‐conditional, and used a 1.5‐T scanner. They also found that battery status, lead threshold, and impedance were not significantly different before and after vasodilator CMR. Furthermore, Miller et al. [25] had 18 CMR‐conditional PPMs or ICDs and two nonconditional ICDs, with all but three patients (three 3‐T conditional devices on 3‐T scanners) performed in 1.5‐T scanners. These studies reported no adverse events. Besides, Motazedian et al. [26] even performed the first safe and successful 3‐T stress perfusion CMR in a patient with an MR‐conditional device.

The safety outcomes and device integrity data are consolidated in Table 3. The absence of documented adverse events in these investigations proves that CMR imaging, especially at higher field strengths, is safe for patients with MR‐conditional devices. While implanting MR‐conditional devices for future MR compatibility in resource‐rich countries is recommended, the limited available evidence suggests scans in patients with non‐MR‐conditional devices may be performed in select cases, though this requires extreme caution due to the lack of manufacturer‐defined safety conditions and a higher potential for artifacts. This body of evidence underscores the safety and effectiveness of vasodilator stress CMR in patients with MR‐conditional devices.

**TABLE 3:** Safety profile and device compatibility.

First author (year)	Device type	Field strength	Population (stress CMR)	Major adverse events	Device parameter changes Post‐CMR	Conclusion on safety	Ref
Klein‐Wiele (2015, 2017)	PPM (conditional)	1.5 T	24, 47	None	No significant change in pacing threshold, impedance, or battery voltage.	Safe.	[11, 12]
Klein‐Wiele (2016)	PPM (conditional)	1.5 T	59	None	No significant change in lead impedance, sensing amplitudes, or battery voltage. A statistically significant decrease in ventricular pacing capture threshold (*p* = 0.027) was noted but is likely not clinically relevant. Atrial threshold unchanged.	Safe and feasible.	[23]
Lindemann (2020)	ICD (45% conditional)	1.5 T	27	None	Battery status, lead threshold, impedance not significantly different.	Safe in mixed conditional/nonconditional cohort.	[24]
Pavon (2022)	PPM and ICD (conditional)	1.5 T	66	None	No significant changes at 1‐year follow‐up.	Safe and feasible.	[17]
Miller (2022)	PPM/ICD (90% conditional)	1.5 T (*n* = 16) 3 T (*n* = 3)	19	One scan aborted (non‐cond. ICD) due to artifact prestress. No clinical adverse event.	Not reported.	Safe for conditional devices. Extreme caution for nonconditional.	[25]
Motazedian (2022)	PPM (conditional)	3 T	1	None	Not reported.	Safe at 3 T in a single case.	[26]
Pezel (2023)	PPM (conditional)	1.5 T	304	None	No significant change.	Safe and feasible.	[27]

*Note:* PPM: permanent pacemaker.

Abbreviation: ICD, implantable cardioverter‐defibrillator.

### 3.5. Device Protocol and Exclusion Criteria

Among the eight included studies, clinicians set the MR‐conditional devices to MR‐safe modes as instructed by manufacturers and reprogrammed them immediately after imaging. Five articles further explained their protocol [11, 12, 17, 25, 27].

Pezel et al. [27] defined their device protocol during stress CMR as set to DOO/VOO for pacemaker‐dependent patients whose heart rate (HR) was below 30 and VVI/DDI for others.

In another study, Pavon et al. [17] utilized continuous pacing in asynchronous mode (at 10 bpm more than resting HR) irrespective of their actual rhythm if > 1% atrial or ventricular pacing was present; VOO mode for continuous high‐grade atrioventricular (AV) block; and DOO mode in intermittent AV block or sick sinus syndrome. They also reported that medical staff performed pre‐CMR programming and continuous electrocardiogram monitoring if an ICD was present.

Furthermore, Klein‐Wiele et al. [11, 12] followed the same protocol in their two studies: no pacing (ODO) in sinus node dysfunction (SND) or atrial fibrillation (AF) with resting HR > 45 bpm; asynchronous atrial stimulation (AOO, 60 bpm) in SND and HR ≤ 45 bpm; asynchronous continuous pacing in permanent or intermittent AV‐block grade 1; VOO at a minimum of 60 bpm or 10 bpm above intrinsic HR (IHR) if sinus rate > 45; DOO at 60 bpm if sinus rate is ≤ 45 bpm; and VOO at 60 bpm in patients with AF and HR ≤ 45 bpm at rest.

Moreover, in their last study [11], Klein‐Wiele et al. performed a test of adenosine infusion at the rate of 140 μg/kg/min in 3 min in patients with intermittent AV block who had preserved AV conduction to predict AV node response to adenosine and determine the device’s mode. If the patient progressed to the 2nd or 3rd AV block, VOO mode at 10 bpm > IHR was activated, and if preserved AV conduction, ODO mode was activated.

In another study, Miller et al. [25] set the device protocol to asynchronous mode with 10 bpm above resting HR or baseline paced rate on the imaging day in patients predicted to necessitate asynchronous pacing during imaging. In their study, 10 out of 20 patients were programmed to asynchronous pacing (one patient in this group had a 0.03% pacing burden), and the rest were deactivated to OOO, OVO, OAO, or ODO programs during the scan (here, seven patients had a pacing burden > 1%).

For ensuring safety, all studies performed stress CMR at least 6 weeks postdevice implantation, and except in the case report by Motazedian et al. [26], stress CMR was performed only after a CXR with no evidence of abandoned, epicardial, or fractured leads. Standard exclusion criteria were as aforementioned in the methods. Other specific exclusion criteria were recent ventricular arrhythmias, inability to use asynchronous pacing in pacing‐dependent patients, unstable congestive heart failure, any known cardiomyopathy and myocarditis, and 2nd or 3rd‐degree AV block (due to agent contraindication) [27]; ejection fraction (EF) < 35% or presence of a tachycardia device [11]; nondiagnostic stress CMR [24, 25]; and an implanted Reveal LINQ Insertable Cardiac Monitor (Medtronic, Dublin, Ireland) [17].

An overview of the various device programming strategies employed across studies is provided in Table 4. These findings underscore the importance of established protocols and careful patient selection in ensuring the accuracy and safety of stress CMR in patients with MR‐conditional devices. Despite the variations in protocols, the consistent use of MR‐safe modes during imaging and the quick reprogramming of the devices afterward have proven to be effective strategies for managing these patients. This body of evidence provides valuable insights for clinicians and researchers in the field, paving the way for future optimized and patient‐centered approaches.

**TABLE 4:** Device protocols during stress CMR.

First author (year)	Underlying rhythm/condition	Recommended device programming mode	Rationale/goal	Ref
Klein‐Wiele (2015)	SND with resting HR > 45 bpm and preserved AV conduction (including AF).	Deactivation/sensing only (ODO).	Allow intrinsic HR response to adenosine. Avoid competitive pacing. Adenosine led to significant HR acceleration (+12.3 bpm), demonstrating sympatho‐excitatory effect overrides bradycardic risk in SND.	[12]
	SND with HR ≤ 45 bpm.	Asynchronous atrial stimulation (AOO at 60 bpm).	Provide atrial pacing support to prevent bradycardia.	
	AV block > 1° (permanent or intermittent) with sinus rate > 45 bpm.	Asynchronous ventricular pacing (VOO at 10 bpm > intrinsic HR, min 60 bpm).	Avoid bradycardia/asystole from adenosine‐induced worsening AV block. Prevent competitive atrial stimulation.	
	AV block > 1° with sinus bradycardia ≤ 45 bpm.	Asynchronous dual‐chamber pacing (DOO at 60 bpm).	Provide continuous dual‐chamber support. Avoid competitive atrial stimulation.	
	AF with resting HR ≤ 45 bpm.	Asynchronous ventricular pacing (VOO at 60 bpm).	Provide ventricular support in bradycardic AF.	

Klein‐Wiele (2017)	SND without AV block and resting HR > 45 bpm.	Deactivation/Sensing only (ODO).	Allow intrinsic HR response to adenosine. The sympatho‐excitatory effect of adenosine leads to significant HR acceleration (*p* < 0.001).	[11]
	SND with HR ≤ 45 bpm.	Asynchronous atrial stimulation (AOO at 60 bpm).	Provide atrial pacing support to prevent bradycardia. Avoids competitive atrial stimulation, which could induce AF.	
	AF with resting HR ≤ 45 bpm.	Asynchronous ventricular pacing (VOO at 60 bpm).	Provide ventricular support in bradycardic AF.	
	Permanent high‐grade AV block (present at time of CMR).	Asynchronous pacing (VOO if SR > 45 bpm, DOO if SR ≤ 45 bpm) at 10 bpm > intrinsic HR (minimum 60 bpm).	Avoid bradycardia/asystole from adenosine‐induced worsening AV block. Prevent competitive atrial stimulation.	
	Intermittent AV block with currently preserved AV conduction.	Perform an adenosine pretest (140 µg/kg/min for 3 min with the device deactivated (ODO)). If 2nd/3rd‐degree AV block develops ⟶ VOO at 10 bpm > intrinsic HR. If conduction preserved ⟶ ODO.	Personalized mode selection based on individual AV node response to adenosine. Identifies patients who can safely have device deactivated vs. those requiring asynchronous support.	

Miller (2022)	Pacing‐dependent patients (e.g., high‐grade AV block, HR < 45 bpm) AND patients with > 1% pacing burden predicted to need support during vasodilator stress.	Asynchronous pacing (VOO, DOO, AOO). Set 10 bpm > presenting rhythm (intrinsic or paced rate) on scan day.	Prevent bradycardia from vasodilator‐induced AV block. Provide continuous pacing support. Empiric asynchronous pacing at this rate was safe, with no competitive pacing or adverse arrhythmic events observed.	[25]

Pavon (2022)	> 1% atrial or ventricular pacing (not necessarily dependent)	Continuous asynchronous pacing (DOO/VOO) at 10 bpm > resting HR.	Preempt any unpredictable pacing inhibition from EMI during scan. Avoid bradycardia/asystole from potential adenosine‐induced AV block in patients with any pacing burden. This universal approach was safe, with no competitive pacing or arrhythmias observed.	[17]
	Continuous high‐grade AV block.	VOO mode.	Provide continuous ventricular support.	
	Intermittent AV block or sick sinus syndrome.	DOO mode.	Provide dual‐chamber asynchronous support.	
	No pacing required (< 1% pacing).	ODO mode (PPM function off).	Allow intrinsic rhythm.	

Pezel (2023)	Pacemaker‐dependent (HR < 30 bpm).	Asynchronous pacing (DOO or VOO).	Ensure continuous pacing support.	[27]
	Nonpacemaker‐dependent.	Nontracking modes (VVI or DDI).	Allow intrinsic HR response with backup.	

*Note:* AV: atrioventricular, EMI, electromagnetic Interference.

Abbreviations: AF, atrial fibrillation; HR, heart rate; SND, sinus node dysfunction; SR, sinus rate.

### 3.6. Vasodilator Choice, Effects, and Safety

A study utilized IV dipyridamole 0.84 mg/kg infused in 3 min in two hundred sixty‐seven patients [27]. One used IV adenosine 140 μg/kg/min over 6 min in one hundred seven [27], five used IV adenosine 140 μg/kg/min over 3 min in sixty‐six [17], fifty‐nine [23], forty‐seven [11], twenty‐four [12], and twelve [25], and a study used IV regadenoson 0.4 mg followed by aminophylline reversal in seven [25] patients.

In this regard, Pezel et al. [27] reported that adenosine/dipyridamole induced no arrhythmia, significant AV node effect, higher AV block, or competitive ventricular stimulation.

Similarly, Klein Wiele et al. [11, 12] showed in their two studies that adenosine infusion was safe and led to neither significant influence on AV node conduction nor higher grades of AV block. Notably, they indicated that after adenosine infusion in patients with (a) permanent or progressive AV block, continuous asynchronous pacing (VOO or DOO mode) did not lead to competitive atrial stimulation or arrhythmia; (b) SND and normal AV conduction, PM deactivation (ODO mode) led to significant HR acceleration (60.1 ± 9.1 to 76.0 ± 9.3 bpm, *p* < 0.001, and by 12.3 ± 8.3 bpm, *p* = 0.001, respectively); (c) sinus rate of 45 bpm, asynchronous atrial stimulation (AOO mode at 60 bpm) led to permanent ventricular capture with no HR acceleration or arrhythmia; and (d) AF (in four patients), ODO mode leads to unchanged HR in three and acceleration from 110 to 122 bpm in one.

In line with them, Miller et al. [25] found that device deactivation led to significant HR acceleration (by 18 bpm, 14–21, and *p* = 0.01), while patients with asynchronous pacing had no considerable HR variability. Besides, diastolic blood pressure (BP) decreased significantly in both patients with deactivated and asynchronous pacing (*p* = 0.04 and 0.01, respectively), but systolic BP decreased significantly only in the first group. This study administered regadenoson instead of adenosine for the first time in stress CMR of patients with pulmonary diseases precluding adenosine use or with single peripheral intravenous access points. In 12 patients receiving adenosine, nine showed positive splenic switch‐off signs, two did not have a visible spleen in the first‐pass perfusion sequences, and one patient with asynchronous pacing had no splenic switch‐off.

Pavon et al. [17] corroborated earlier findings about HR acceleration of nonpaced patients. However, diastolic BP dropped significantly in paced patients (from 76.0 ± 13.6 mmHg to 68.7 ± 12.5 mmHg, *p* = 0.007), unlike the stabilized BP in nonpaced patients. In this study, 78% of 50 paced patients had splenic switch‐off, and 36% had mild to moderate respiratory responses to adenosine. The differential hemodynamic responses based on pacing mode are synthesized in Table 5.

**TABLE 5:** Hemodynamic changes.

Hemodynamic parameter	Deactivation/sensing mode (ODO, OVO, etc.)	Asynchronous pacing mode (VOO, DOO, etc.)	Summary and clinical implication
HR response	Significant increase. • Klein‐Wiele (2015): +12.3 ± 8.3 bpm (*p* = 0.001) [12]. • Klein‐Wiele (2017): SND: 60.1 ± 9.1 to 76.0 ± 9.3 bpm (*p* < 0.001). Intermittent AV‐block (preserved conduction): 67.6 ± 9.1 to 77.6 ± 11.3 bpm [11]. • Miller (2022): median +18 bpm [IQR 14–21] (*p* = 0.01) [25]. • Pavon (2022): HR stable (nonpaced patients) [17].	No significant change. • Klein‐Wiele (2015): remained constant in AV‐block patients [12]. • Klein‐Wiele (2017): remained constant [11]. • Miller (2022): no change with stress [25].	HR acceleration is a physiologic response to vasodilators seen only when intrinsic conduction is allowed. Its absence in asynchronous pacing can be a confounder.
SBP response	Significant decrease. • Miller (2022): significant decrease (*p* = 0.04) [25].	No significant change/stabilized. • Miller (2022): no significant change [25]. • Pavon (2022): no change [17].	SBP drop is part of the vasodilatory effect. Asynchronous pacing may blunt this response, potentially affecting afterload and perfusion assessment.
DBP response	Significant decrease. • Miller (2022): significant decrease (*p* = 0.04) [25].	Significant decrease. • Miller (2022): significant decrease (*p* = 0.01) [25]. • Pavon (2022): significant decrease (*p* = 0.007) [17].	DBP decreases regardless of pacing mode, indicating a consistent systemic vasodilatory effect of the stress agent.
Stress efficacy (splenic switch‐off)	Reported as present in studies (e.g., Miller (2022): 9/12 patients with adenosine) [25].	May be attenuated. • Miller (2022): One patient with async. pacing lacked switch‐off [25]. • Pavon (2022): 78% of paced patients had switch‐off [17].	Splenic switch‐off is a marker of adequate stress. Asynchronous pacing may be associated with a lower rate of adequate switch‐off, suggesting a blunted hemodynamic response.

Abbreviations: DBP, diastolic blood pressure; HR, heart rate; SBP, systolic blood pressure.

Research has demonstrated that pharmacological stress agents, including adenosine, dipyridamole, and regadenoson, are safe and effective for inducing stress CMR. Notably, arrhythmias, effects on the AV node, and higher grades of AV block were not observed as significant adverse outcomes. In the context of stress CMR, patients with asynchronous pacing did not exhibit significant changes in HR variability. Conversely, patients whose devices were deactivated experienced an increase in HR. A notable decrease in systolic BP was observed exclusively in the group with deactivated devices. A drop in diastolic BP was recorded in both the deactivated and asynchronously paced groups.

Moreover, regadenoson presents a viable alternative to adenosine for individuals with respiratory conditions or limited intravenous access. Observing a positive splenic switch‐off further underscores these stress agents’ successful elicitation of physiological responses. In conclusion, considering these limited studies, this review affirms pharmacological stress agents’ safe and reliable use for CMR imaging in patients with CIEDs.

### 3.7. Clinical Outcomes

In a study by Pezel et al. [27], 273 out of 304 patients completed a median follow‐up of 7.1 years [IQR: 5.4–7.5]. Ischemia and LGE were detected in 54 and 81 patients, respectively. Among 54 patients with ischemia (2.5 ± 1.8 segments out of 17), 48 underwent coronary angiography, and 45 had a CMR‐related coronary revascularization. In a multivariable model adjusted for hypertension, diabetes, and known CAD, both ischemia and LGE were independent predictors of MACE, defined as cardiovascular mortality and nonfatal myocardial infarction (hazard ratio: 5.29 [95% CI: 2.64–14.54] and hazard ratio: 2.96 [95% CI: 2.22–4.00], respectively, both *p* < 0.001).

Besides, in an earlier long‐term study by Pezel et al. [16], 203 out of 224 patients who underwent stress CMR completed the follow‐up (median: 7 years [IQR: 5.2–7.3]), during which 23 MACE, defined as cardiovascular mortality and nonfatal myocardial infarction, occurred. Inducible ischemia was present (mean extent: 2.5 ± 1.8 segments out of 17) in 41 patients (20.2%). Subsequently, 35 had coronary angiography, and 31 underwent CMR‐related coronary revascularization. Also, 70 patients (34.5%) had LGE. The presence and extent of inducible ischemia and LGE were independent predictors of MACE (hazard ratio: 5.24; 95% CI: 2.61–14.40, hazard ratio: 1.71; 95% CI: 1.44–2.03) and LGE (hazard ratio: 2.98; 95% CI: 2.25–4.02, respectively, for all: *p* < 0.001).

Long‐term follow‐ups revealed that inducible ischemia and LGE could independently predict MACE. Lastly, positive adenosine CMR tests were confirmed with coronary angiography, leading to CMR‐related coronary revascularization in several cases. These findings underscore the clinical significance of stress CMR in managing cardiac patients.

### 3.8. Limitations

Despite the significant insights gained from this systematic review, several knowledge gaps have been identified that need to be covered in future research. A major limitation of this review is the severe paucity of data concerning non‐MR‐conditional devices. The included studies mainly focused on MR‐conditional devices, meaning the conclusions of this review are therefore primarily applicable to that population. The extremely limited evidence on non‐MR‐conditional devices precludes definitive statements on their safety or diagnostic utility for stress CMR, highlighting a critical area for future investigation. Also, the assessment for LGE was limited by the non‐utilization of a wide‐band sequence, which is the standard for reducing artifacts in patients with ICDs. Moreover, research on stress CMR in patients with cardiac implantable CIEDs is scarce, underscoring the importance of conducting larger‐scale investigations with systematic analyses to assess the applicability of this valuable modality in this specific population. Furthermore, enhanced patient education regarding procedural safety, device‐specific considerations, and postprocedural monitoring is imperative to support informed decision‐making and optimize clinical outcomes in this vulnerable cohort [30].

## 4. Conclusion

This systematic review synthesizes evidence from eight studies involving approximately 500 patients, providing a comprehensive assessment of stress CMR in patients with CIEDs. The aggregated data confirm that vasodilator stress CMR is safe, feasible, and provides diagnostic image quality in patients with MR‐conditional devices when performed according to established protocols. Clinicians should consider device laterality when anticipating image quality and employ specific programming strategies to maintain safety during pharmacological stress. While there have been improvements in image quality, challenges remain. It is crucial to emphasize that these findings and recommendations are based on evidence from limited studies of MR‐conditional devices at mostly 1.5‐T scanners; the applicability to patients with non‐MR‐conditional devices or using 3‐T scanners remains uncertain and warrants dedicated study. Overcoming the current challenges will require targeted technological development alongside dedicated research in three key areas. First, studies must directly compare the hemodynamic profiles of different pharmacological stressors in patients with CIEDs across various pacing modes. Second, longitudinal investigations are needed to establish robust, long‐term prognostic data for this population and to identify the CMR parameters that best predict clinical outcomes. Finally, technological innovation, particularly in artifact reduction sequences and compatibility with higher‐field‐strength scanners, is essential to expand diagnostic capability. Addressing these specific knowledge gaps through focused research will be fundamental to optimizing protocols and ultimately improving patient care.

## Author Contributions

N.O., M.R.K., and G.H.: conceptualization, project administration, methodology, and writing–review and editing; S.Z., P.B., A.B., R.R., M.F., and E.M.: investigation, data curation, writing–original draft, writing–review and editing, and visualization; F.A., A.F., H.P., and H.H.: supervision, methodology, and validation.

## Funding

The authors declare that no funds, grants, or other support were received during the preparation of this manuscript.

## Disclosure

All authors agree to be accountable for the content and conclusions of the article.

## Ethics Statement

This study was performed in line with the principles of the Declaration of Helsinki. The study was registered with the PROSPERO under the ID CRD42023457308. A statement of ethics is not applicable because this study is based exclusively on published literature.

## Consent

The authors have nothing to report.

## Conflicts of Interest

The authors declare no conflicts of interest.

## Supporting Information

Additional supporting information can be found online in the Supporting Information section.

## Supporting information


**Supporting Information 1** Supporting Table 1. PRISMA checklist.

**Supporting Information 2** Supporting Table 2. Search strategy.

**Supporting Information 3** Supporting Table 3. The quality assessment of included studies.

## Data Availability

Data sharing is not applicable to this article as no datasets were generated or analyzed during the current study.
