# Supplementary material for: Applicability of Stress Cardiac Magnetic Resonance Imaging in Patients With Cardiac Implantable Devices: A Systematic Review
Source: Cardiol Res Pract. 2026 Jun 28;2026:3131535. doi: 10.1155/crp/3131535 (PMC13310391; doi:10.1155/crp/3131535)
Supplement: Supplementary file 2 — Supporting Information 2 Supporting Table 2. Search strategy. [file CRP-2026-3131535-s002.docx]

**Supplemental Table S2.** Search strategy.

(“Artificial Pacemaker” OR “Artificial Pacemakers” OR “Artificial Cardiac Pacemaker” OR “Artificial Cardiac Pacemakers” OR “Implantable Defibrillator” OR “Implantable Defibrillators” OR “Implantable Cardioverter Defibrillator” OR “Implantable Cardioverter Defibrillators” OR “Implantable Cardioverter-Defibrillator” OR “Implantable Cardioverter-Defibrillators” OR “Defibrillator” OR “Electric Shock Cardiac Stimulators” OR “Automated External Defibrillators” OR “Automated External Defibrillator” OR “External Defibrillator” OR “External Defibrillators” OR “cardiac implantable electronic devices” OR “cardiac implantable electronic device” OR “implantable electronic devices” OR “implantable electronic device” OR “MR Conditional pacemaker” OR “MR Conditional pacemakers” OR “MR-Conditional pacemaker” OR “MR-Conditional pacemakers” OR “Magnetic resonance conditional pacemaker” OR “Magnetic resonance conditional pacemakers” OR "Cardiac rhythm management device") AND (Stress OR Perfusion OR "Vasodilator agent" OR Vasodilator OR Vasodilators OR Adenosine OR Regadenoson OR dipyridamole) AND (“Cine Magnetic Resonance Imaging” OR “Cine MRI” OR “Cine MRIs” OR “CMR” OR “CMRs” OR "Cardiovascular magnetic resonance" OR “Cardiac CMR” OR “cardiac magnetic resonance imaging” OR “Feature tracking cardiac magnetic resonance” OR "Cardiac magnetic resonance feature tracking")
