# Supplementary material for: Applicability of Stress Cardiac Magnetic Resonance Imaging in Patients With Cardiac Implantable Devices: A Systematic Review
Source: Cardiol Res Pract. 2026 Jun 28;2026:3131535. doi: 10.1155/crp/3131535 (PMC13310391; doi:10.1155/crp/3131535)
Supplement: Supplementary file 3 — Supporting Information 3 Supporting Table 3. The quality assessment of included studies. [file CRP-2026-3131535-s003.docx]

**Supplemental Table S3.** The quality assessment of included studies.

| First author | The Newcastle- Ottawa quality assessment | | | Final score | Reference |
| --- | --- | --- | --- | --- | --- |
|  | Selection  (Maximum 5 score) | Comparability  (Maximum 2 score) | Outcome  (Maximum 3 score) |  |  |
| Klein-Wiele | **** | ** | *** | 9 | [12] |
| Klein-Wiele | **** | - | *** | 7 | [24] |
| Klein-Wiele | **** | - | *** | 7 | [11] |
| Lindemann | **** | - | *** | 7 | [28] |
| Miller | ***** | ** | *** | 10 | [26] |
| Pavon | **** | - | *** | 7 | [17] |
| Pezel | **** | ** | *** | 9 | [25] |
|  | **JBI Critical Appraisal Checklist for Case Reports (Maximum 8 score)** | | |  |  |
| Motazedian | - | - | - | 8 | [27] |
